# Medium density EMG armband for gesture recognition

**DOI:** 10.3389/fnbot.2025.1531815

**Published:** 2025-04-30

**Authors:** Eisa Aghchehli, Milad Jabbari, Chenfei Ma, Matthew Dyson, Kianoush Nazarpour

**Affiliations:** ^1^School of Engineering, Newcastle University, Newcastle upon Tyne, United Kingdom; ^2^School of Informatics, The University of Edinburgh, Edinburgh, United Kingdom

**Keywords:** medium-density, myoelectric control, gesture recognition, temporal neural network, machine learning

## Abstract

Electromyography (EMG) systems are essential for the advancement of neuroprosthetics and human-machine interfaces. However, the gap between low-density and high-density systems poses challenges to researchers in experiment design and knowledge transfer. Medium-density surface EMG systems offer a balanced alternative, providing greater spatial resolution than low-density systems while avoiding the complexity and cost of high-density arrays. In this study, we developed a research-friendly medium-density EMG system and evaluated its performance with eleven volunteers performing grasping tasks. To enhance decoding accuracy, we introduced a novel spatio-temporal convolutional neural network that integrates spatial information from additional EMG sensors with temporal dynamics. The results show that medium-density EMG sensors significantly improve classification accuracy compared to low-density systems while maintaining the same footprint. Furthermore, the proposed neural network outperforms traditional gesture decoding approaches. This work highlights the potential of medium-density EMG systems as a practical and effective solution, bridging the gap between low- and high-density systems. These findings pave the way for broader adoption in research and potential clinical applications.

## 1 Introduction

The need for intuitive and accurate myoelectric control systems has grown rapidly with the increase in the use of prosthetic hands in rehabilitation, assistive devices, and gesture tracking interfaces for the Metaverse (Vaca Benitez et al., 2013; Toledo-Peral et al., 2022; labs at Reality Labs et al., 2024).

For millions of people around the world living with upper-limb differences or amputations, prosthetic hands offer a way to regain dexterity and perform daily tasks. However, prosthetic hand technology still faces significant challenges (Mendez et al., 2021). Despite advances in size, weight, and functional capabilities, the control mechanisms of prosthetic hands remain limited, making it difficult for users to achieve precise and natural movements. Myoelectric control, a widely used method for controlling these devices, involves decoding electrical signals generated by muscle activity. Conventional low-density electromyography (EMG) systems typically use only a few electrodes (Resnik et al., 2018), resulting in limited spatial resolution and accuracy. Although high-density EMG systems offer better spatial resolution, their large footprint and requirement for precise positioning can be cumbersome, particularly in wearable applications (labs at Reality Labs et al., 2024; Jiang et al., 2021).

EMG-based systems traditionally employ bipolar electrodes to record signals from two antagonistic muscles in the forearm, allowing basic control over one degree of freedom in prosthetic hands (Geethanjali, 2016). Users modulate their muscle activity to exceed a threshold on one electrode, moving prosthetic hands in a particular direction. However, this approach restricts functionality to one degree of freedom at a time. Pattern recognition algorithms have improved the functionality of prosthetic hands by decoding various grasps directly from EMG signals, enhancing both dexterity and intuitive use (Parajuli et al., 2019; Jiang et al., 2024). However, this method usually requires up to eight bipolar electrodes and involves a complex calibration phase to map each grasp type to the user's unique muscle activity patterns. Although these approaches have achieved promising research results, their practical implementation is hindered by limitations such as the complexity of setup, the reliance on experienced personnel for precise electrode placement, and the time-consuming calibration (Mendez et al., 2021).

Recent advances in machine learning, particularly deep learning, have offered new approaches for decoding motor intentions from EMG data. Convolutional neural networks, originally popularized in computer vision, have demonstrated high potential in EMG applications, improving accuracy and robustness in grasp classification. Their capacity to learn features directly from raw signals enables them to outperform traditional machine learning models in several studies. However, even with their powerful feature extraction capabilities, conventional EMG systems still rely on carefully placed bipolar electrodes for reliable classification, which limits their accessibility and practicality for patients. This dependency creates challenges, especially for users with amputations resulting from traumatic events, which can alter the position or structure of muscles in the residual limb.

To overcome these limitations, researchers have investigated high-density EMG, which uses electrode grids to record muscle activity in high spatial detail and captures two-dimensional images of muscle activation. Although promising results can be achieved (Jiang et al., 2021), the high electrode count and spatial resolution make it unsuitable for wearable applications due to the large footprint of the system and the requirement for gel-based electrodes, which can be uncomfortable for daily wear. A trade-off between low- and high-density EMG is medium-density EMG, which uses fewer electrodes with increased spatial coverage but preserves the compactness of low-density systems. A medium-density EMG system can be in the form of an armband (Rawat et al., 2016) and, therefore does not require precise electrode placement. It would be easier to configure and can be embedded in the prosthesis socket or in a wearable device for practical, real-life applications.

In this work, we introduce a medium-density EMG system that addresses current limitations in myoelectric control by incorporating 21 digital electrodes within a single, compact armband. This armband significantly improves decoding performance while maintaining the small footprint typical of low-density systems. Additionally, we propose a novel time-domain-based deep neural network architecture based on the Temporal Convolutional Network (TCN) for the classification of EMG signals. Our study presents the first controlled comparison of medium- and low-density EMG systems. We evaluated their respective classification accuracies in identical experimental conditions on simultaneously recorded data.

## 2 Methods

We first describe the development of a medium-density EMG armband. We then focus on quantifying the impact of additional EMG sensing units on gesture decoding performance.

### 2.1 Hardware design

**Figure 1:**
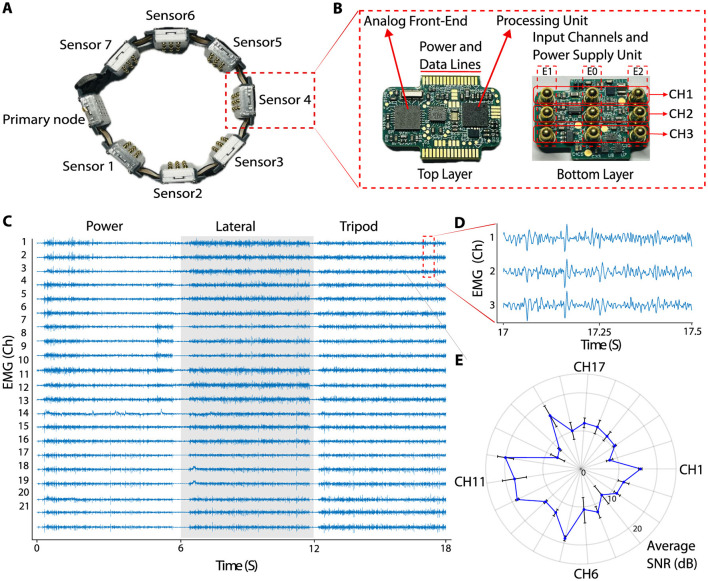
**(A)** Prototyped medium-density armband, with one primary node and seven sensor nodes. **(B)** Assembled PCB of the sensor. Top layer accommodate analog front-end and processing units, and the bottom layer consists of parts for power supply and the input channel contacts. **(C)** 18 seconds of data recorded (6 seconds for each grasp, eliminating the rest period in between two movements) during three different grasps using the medium-density armband. **(D)** Zoomed-in view of data recorded from channels 1, 2, and 3, between times 17 and 17.5 seconds data showing small differences between channels despite close proximity. **(E)** Average SNR plot for a sample 30-second EMG data recorded during a grasp for all 21 channels.

The development of the hardware for the medium-density EMG armband was guided by design principles established in our previous research (Aghchehli et al., 2024), which laid the foundation for a modular and scalable digital EMG recording system. The prototyped medium-density EMG system is shown in Figure 1A. Each sensor module includes an analog front-end (AFE) for signal acquisition and an EMG signal processing subsystem. The AFE was built on an ADS1293 platform (Texas Instruments, USA) (Texas Instruments, 2013), which features a fixed-gain pre-amplifier, a 24-bit analog-to-digital converter across three channels, and an integrated electromagnetic interference (EMI) filter. As shown in Figure 1B, each channel interfaces with two active electrodes and a reference electrode (E1, E2, and E0), performing differential recordings with the reference attached to the system ground. The details of the system are listed in Table 1.

**Table 1:** System details per sensor.

Number of recording channels per sensor	3
Sampling rate	1,067 *SPS*
Digitization resolution	24 *Bits*
Bandwidth	30–350 *Hz*
Input range (Fixed gain= 3.5x)	–400 *mV* to +400 *mV*
AFE Input referred Noise	2.25 μ*V*_*p*−*p*_
AFE Common Mode Rejection Ratio (CMRR)	107 dB
Maximum SNR	20 dB
Power consumption	50 mW
PCB layers	8
PCB dimensions	24 × 18 *mm*

The EMG processing unit was powered by a 32-bit ARM Cortex-M4 microcontroller (STM32L433RCI3, STMicroelectronics), connected to the analog front-end via a 16 MHz Serial Peripheral Interface (SPI) interface. It sampled the EMG signals at 1,067 Hz. Signal filtering was performed using a second-order infinite-impulse response (IIR) Butterworth bandpass filter (30–350 Hz) to remove undesired spectral components, and a notch filter (50/60 Hz) to eliminate power-line interference.

To prevent electrical hazards, meeting the International Electrotechnical Commission (IEC) 60601-1 standard for medical electrical equipment safety, each input channel and AFE were protected with a 10kΩ resistor. The electrode contacts were built with commercially available surface mount spring-loaded contacts (Beryllium Copper), which are compliant with the Restriction of Hazardous Substances directive. The resistance of each contact is about 30 mΩ according to the datasheet (Mill-Max Manufacturing Corporation, 2024). The system was housed in a 3D-printed case designed in Fusion360 and printed using PLA with a Bambu Lab P1S printer.

The medium-density EMG armband consists of two types of nodes: one primary node and many sensor nodes. The primary node functions as the network controller and serves as a bridge between the sensor network and the external device; enabling data transmission and analysis. The nodes are interconnected using a 32MHz SPI link. The primary node transfers data which are collected from all sensors, through USART to a computer using USB to a serial cable (DSD TECH, 2024). The nodes are connected using a 16-way, 0.5mm pitch flex-PCB cable, allowing the armband to conform comfortably around the forearm. The system is powered via USB 2.0, and each node containing an internal power management unit that supplies the necessary power to both the AFE and the EMG processing unit.

#### 2.1.1 Signal to noise ratio calculation

The quantitative assessment of EMG signals captured using medium-density EMG sensors under dynamic conditions was performed to evaluate sensor quality, which is crucial for various applications, including pattern recognition-based systems. The average Signal-to-Noise Ratio (SNR) for each channel was calculated using the algorithm outlined in Agostini and Knaflitz (2011) and Aghchehli et al. (2024). The EMG signals were divided into ON and OFF states to represent periods of activity and rest. We calculated the variance of the noise during the OFF state and the variance of the signal during the ON state. If *N* represents the number of OFF states, the background noise *e*_*n*_ can be defined with $e_{n}=\frac{1}{N}\sum_{i=1}^{N}\sigma_{n_{i}}$ where *e*_*n*_ represents the baseline noise level, σ_*n*_*i*__ is the standard deviation of the *i*-th OFF state, and *N* is the total number of data points in the OFF state. To distinguish between ON and OFF states, we applied the double-threshold method described by Agostini and Knaflitz (2011). The EMG signals were first rectified and then two thresholds were set: the Lower Threshold (LT), defined just above the baseline noise, was calculated as a multiple of the baseline noise standard deviation, while the Upper Threshold (UT), set higher, confirmed muscle activation, also based on a larger multiple of the baseline noise's standard deviation. The calculations are as follows: $\mu_{n}=\frac{1}{N}\sum_{i=1}^{N}x_{i}$ where μ_*n*_ represents the mean of the baseline noise, *N* is the number of data points in the baseline segment, and *x*_*i*_ represents each individual data point.

Concretely:


$$\begin{array}{l} LT=\mu_{n}+k_{1}\sigma_{n}\\ UT=\mu_{n}+k_{2}\sigma_{n} \end{array}$$

Where *LT* is the lower threshold, *UT* is the upper threshold, and *k*_1_ and *k*_2_ are constants that set the sensitivity of the thresholds, with *k*_1_ < *k*_2_. To avoid misinterpreting brief fluctuations as transitions between ON and OFF states, signals above *LT* were tracked for *m* consecutive activation samples. This approach rejected variations in the detector output shorter than *m* samples. For each consecutive ON and OFF state, the mean variance was calculated and used to compute the SNR, with:


$$\begin{array}{l} SNR=10\log_{10}\left(\frac{\sigma_{s}^{2}}{\sigma_{n}^{2}}-1\right), \end{array}$$

where $\sigma_{s}^{2}$ and $\sigma_{n}^{2}$ are the variances of the ON and OFF states, respectively.

### 2.2 Ethical approval

This study was approved by the local ethics committee of Newcastle University (reference number: 20-DYS-050). Eleven participants (age 19 to 43, 2 women) were recruited. All participants signed an informed consent form before participating in the experiment.

### 2.3 Experiment design

**Figure 2:**
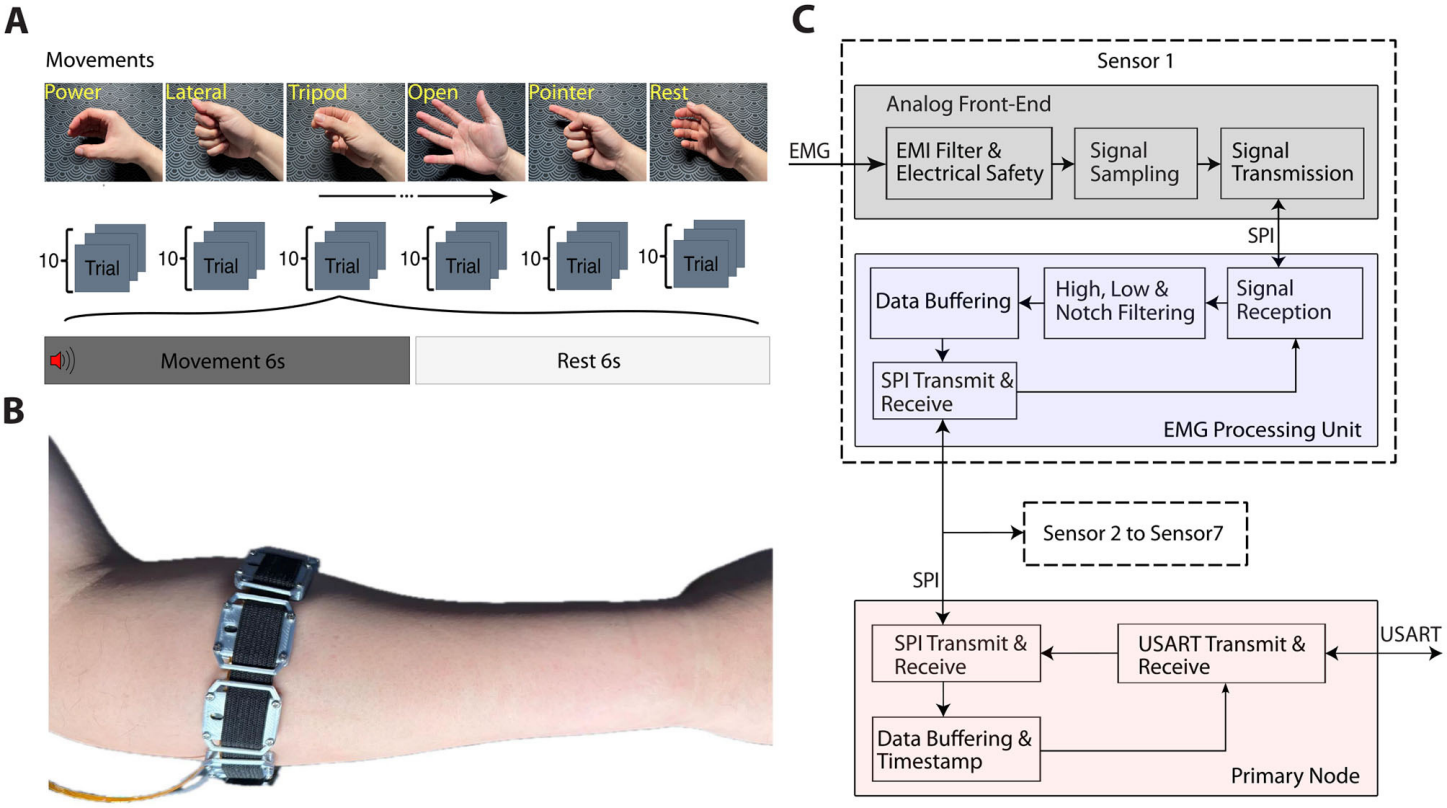
**(A)** The experiment protocol and movement examples. **(B)** A participant wearing the armband on the right forearm. **(C)** EMG signal pipeline, including all utilized built-in blocks within the ADS1293 chip, including filtering for electromagnetic interference rejection, and filtering for anti-aliasing.

In this study, participants performed six common functional movements: power grip, lateral pinch, tripod grip, pointer (extension), opening of the hand, and rest. Each movement was repeated 10 times for a total of 60 trials, as shown in Figure 2A. During each trial, subjects followed a structured protocol in which they engaged in the designated movement for 6 seconds, immediately followed by a relaxation period of 6 seconds to ensure adequate muscle recovery and reduce fatigue. This standardized timing allowed for consistent data capture across movements and ensured that the neural network received well-distributed input samples for training and comparison. Furthermore, the combination of various grip types and rest intervals helped capture a wide range of functional muscle activity that offered a comprehensive data set to test the ability of medium-density electrodes in decoding various movement patterns.

Movement labels were automatically generated for subjects to follow and saved alongside the recordings using self-developed software on the Axopy (Lyons and Margolis, 2019) platform. It is performed as our experimental task generates the label by itself and marks it down automatically. At the same time, the task instructs the desired movement to the subjects and labels the simultaneously recorded data. This software ran on a DELL Latitude 5431 laptop equipped with a 12th Gen Intel(R) Core i7-1270P, 2.2GHz CPU, and 32GB of memory, ensuring smooth processing and data storage. This setup allowed for the precise synchronization of movement labels with the corresponding EMG data and provided a reliable basis for further analysis and neural network training. The automatic labeling feature streamlined the data acquisition process, enhancing the accuracy and efficiency of capturing labeled movement data to decode performance assessments.

Seven sensor nodes, each comprising three EMG channels, were evenly placed around the forearm approximately 4 cm below the elbow to provide equal spacing between blocks to allow consistent signal capture across the forearm's musculature. Figure 2B illustrates the positioning of the electrode and provides a visual reference for the experimental setup and layout of the electrode.

### 2.4 Signal analysis

EMG data passed through a pipeline of signal conditioning and analysis, as detailed in Figure 2C. Hardware components of the pipeline were described earlier and in Table 1.

As for the software components, we conjectured that the medium-density arrangement would lend itself well to demonstrate the additional benefits of adopting spatio-temporal based descriptor features introduced in Samuel et al. (2019). We implemented the six proposed features, referred to as spatio-temporal-based feature sets. The integral square descriptor of the signal, representing its power, is calculated by summing the squared magnitudes of the signal values over a given window. The second and third features were derived from the difference between the integral square descriptor and the normalized root-square coefficients of the first and second differential derivatives. The fourth feature estimates muscle contraction force for a specific gesture. This is achieved using a variant of the non-linearized form of the log-detector presented in Tkach et al. (2010). For the fifth feature, the mean value of the square root of the given window as a temporal representation of the muscle activity is calculated (Samuel et al., 2018). Finally, mean derivatives of the higher order moments were adopted from Al-Timemy et al. (2015). Table 2 presents the equations used to extract these features. In the table, *x* represents a window of the signal, *n* denotes the window length, and *DD*_*x*1_ and *DD*_*x*2_ correspond to the first and second differential derivatives of the signal, respectively. Furthermore, *RSC*_*x*_ represents root-square coefficient of the signal *x*. We used an overlapping segmentation scheme with a window length of 281.1 ms and an incremental step of 9.3 ms to extract features.

**Table 2:** Equations of the six extracted features used in this study.

*ISDsig*	$\sum_{i=0}^{n-1}{x\left[i\right]}^{2}$
Difference between *ISDsig* and normalized *RSC*_*x*1_	$\sum_{i=0}^{n-1}{x\left[i\right]}^{2}-\frac{1}{\theta }\sum_{i=0}^{n-1}{DD_{x1}\left[i\right]}^{2}$
Difference between *ISDsig* and normalized *RSC*_*x*2_	$\sum_{i=0}^{n-1}{x\left[i\right]}^{2}-\frac{1}{\theta }\sum_{i=0}^{n-1}{DD_{x2}\left[i\right]}^{2}$
Mean logarithm kernel	$abs\left(e^{\frac{1}{n}\sum_{i=0}^{n-1}log\left(x\left[i\right]\right)}\right)$
Mean value of the square root	$\frac{1}{n}\sum_{i=0}^{n-1}{x\left[i\right]}^{1/2}$
Mean derivative of the higher order moments	$\frac{1}{n}\sum_{i=0}^{n-1}DD_{x2}\left[i\right]$

In the equations, *x*, *n*, *DD*_*x*1_, and *DD*_*x*2_ represent a window of the signal, the window length, and the first and the second differential derivatives of the signal, respectively. *RSC*_*x*_ denotes root-square coefficient of the signal *x*.

### 2.5 Neural network design

Temporal Convolutional Networks (TCN), as a recently emerged class of deep learning models, have proven their superiority against conventional recurrent networks in many time-series analyses, sequence modeling tasks, and EMG-based hand gesture classification (Lea et al., 2017; Bai et al., 2018; Betthauser et al., 2019; Zanghieri et al., 2019; Jabbari et al., 2024, 2021). The dilated structure of causal one-dimensional convolutional operations along the time dimension makes them computationally efficient and suitable for real-time on-board implementation (Cote-Allard et al., 2021). However, most previous work has implemented the TCN structure in a stacked-layer framework, using purely temporal networks in a straightforward manner (Zanghieri et al., 2019). This approach lacks spatial representation of the signals, as the model focuses solely on the temporal aspect in each layer. Therefore, such models can be regarded as a vanilla TCN structure. To address the spatial aspect of the signal in addition to temporal considerations, most spatio-temporal deep models use independent spatial and temporal blocks in a cascaded framework (Jabbari and Nazarpour, 2024; Xia et al., 2018; Hu et al., 2018; Wu et al., 2018; Ma and Nazarpour, 2024).

**Figure 3:**
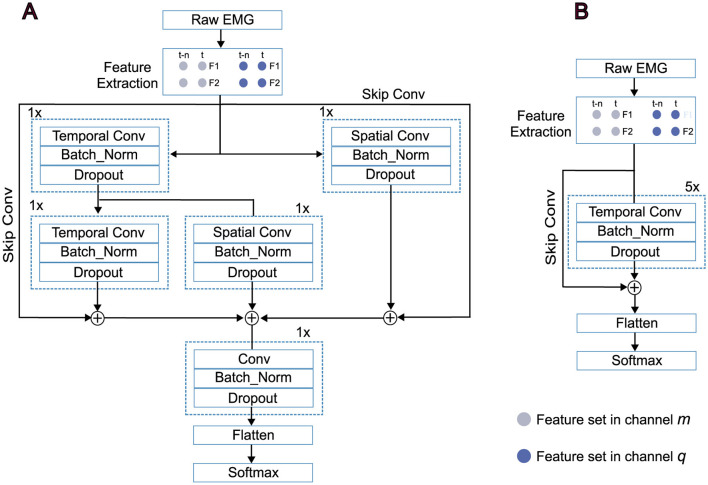
Schematic representations of the spatial-temporal convolutional network. **(A)** The vanilla TCN. **(B)** Segmented raw EMG signals are used to extract features and are then fed into the models based on the deployed configurations. Each dilated convolutional layer is followed by batch normalization and dropout layers.

In this study, we implemented a novel spatio-temporal convolutional network capable of performing spatial and temporal convolutional operations simultaneously. In this structure, each input sample at time *t* is not only convolved to the sample at *t* − *n*, but is also convolved to the input at the same time *t* from other channels. Therefore, this structure can be regarded as a simultaneous spatio-temporal convolutional network. A schematic block diagram of the proposed architecture is shown in Figure 3A. As the schematic diagram illustrates, we present a simple scenario with two features from two time steps, which can be extended to six features and a broader range of time steps. For two features, *F*1 and *F*2, from two channels at two time steps t and *t*_*n*_, the first stage, as shown, applies temporal and spatial convolutions on the right and left, respectively. For the output at time step *t*, the temporal connections are realized by convolving features of channel *m* and *q*, *F*1 and *F*2, from *t* and *t*_*n*_, separately. In parallel, in the spatial convolutional layer, the spatial output is achieved by convolution between *F*1 and *F*2 from channel *m* and *q* at time step *t*. Therefore, features *F*1 and *F*2 from channel *m* at time step *t* not only are connected to features *F*1 and *F*2 from the same channel at time step *t* − *n*, but also are connected to features *F*1 and *F*2 from channel *q*, too. The same approach is applied for features *F*1 and *F*2 from channel *q*. Each convolutional layer is followed by a batch normalization and dropout layer to prevent overfitting, and the final decision is made by a softmax layer. In addition to the proposed spatial-temporal convolutional network, we implemented two baseline approaches for benchmarking. A vanilla TCN architecture (Figure 3B), which applies temporal convolutions purely along the time dimension, and a traditional linear discriminant analysis (LDA) method.

### 2.6 Explainability with saliency maps

We employed the saliency maps method to visually explain how the proposed model extracts spatio-temporal components from the input data. The concept comes from computer vision for image classification, and the main purpose of the method is to show the importance of each *input pixel* when output is generated with an opaque machine learning model (Meng et al., 2023). Each input pixel is assigned an importance score, visualizing the degree of contribution it has to the final output of the model. Inspired by the saliency map in image classification, features of each channel at every time step are considered as a spatio-temporal pixel in a time series classification problem (Crabbé and Van Der Schaar, 2021; Ismail et al., 2020; Yan et al., 2021). We implemented the saliency map method using the *keras.vis* library as described in Simonyan (2013). Taking into account a specific input **I**_0_ and class *c*, as well as the class score function *S*_*c*_(**I**_0_), the purpose is to illustrate the contribution of each channel-time component of **I**_0_ based on its influence on the score *S*_*c*_(**I**_0_). If we consider a linear score for the class *c*, *S*_*c*_(**I**) can be written as:


$$\begin{array}{l} S_{c}\left(\text{I}\right)={\text{w}}_{c}^{T}\text{i}+b_{c} \end{array}$$

Where **i** is the vectorized version of **I** and the magnitude of elements of **w** describes the importance of the corresponding spatio-temporal component of input **I** for the class *c*. In the proposed spatio-temporal model the class score *S*_*c*_(**I**) is a non-linear function of **I**. However, *S*_*c*_(**I**) can be approximated by a first-order Taylor expansion as:


$$\begin{array}{l} S_{c}\left(\text{I}\right)\approx {\text{w}}^{T}\text{i}+b \end{array}$$

Therefore, the main concept behind the idea is to calculate the gradient of the classification score *S*_*c*_ with respect to the input **I** at the spatio-temporal component of **I**_0_. The input shape of deep learning models is structured as [samples, time_steps, channels, features]. For each sample, the score array generated by the Saliency non-linear map follows the shape [time_step, channel_score, feature_score]. In our case, with time_steps, channels, and features being 60, 21, and 6, respectively, the score array takes the form [60, 21, 6]. Since our focus is on investigating the contribution of each channel rather than individual features, we calculated the average score values along the feature dimension. As a result, the final score array for each gesture is [60, 21]. The utilized Saliency method computes gradient-based saliency maps (Meng et al., 2023).

### 2.7 Statistical analysis

To investigate the statistical significance of the achieved results, for the paired comparisons, we utilized the Wilcoxon Signed-Rank test, which is suitable for dependent samples as a non-parametric method. In addition, the Friedman test was deployed to compare multiple conditions. In cases with a significant statistical difference, we used post-hoc pairwise comparisons using the Nemenyi test. All statistical analyses were run using the scipy.stats and scikit-posthocs packages in Python.

## 3 Results

To evaluate SNR in all 21 channels, an additional participant (male, 30 years old) wore the armband and performed a power grasp for one second, followed by a one-second rest period, while standing in front of a screen. The subject was asked to perform the activity to the extent that was comfortable over the course of the data recording. This activity cycle was repeated for a total recording duration of 30 seconds. SNR calculations were performed for each ON and OFF state as described above, and then the average SNR was calculated for each channel over the 30-second recording period. Figure 1C shows an example of 18 seconds of data recorded (6 seconds for each grasp, eliminating the rest period in between two movements) during three different grasps using the medium-density armband. Figure 1D shows the close-up view of the data from channel 1, 2 and 3, between times 17 and 17.5 seconds. Despite the close distance between input contacts, there are small differences between each channel's signals. Figure 1E presents the average SNR for each channel.

**Figure 4:**
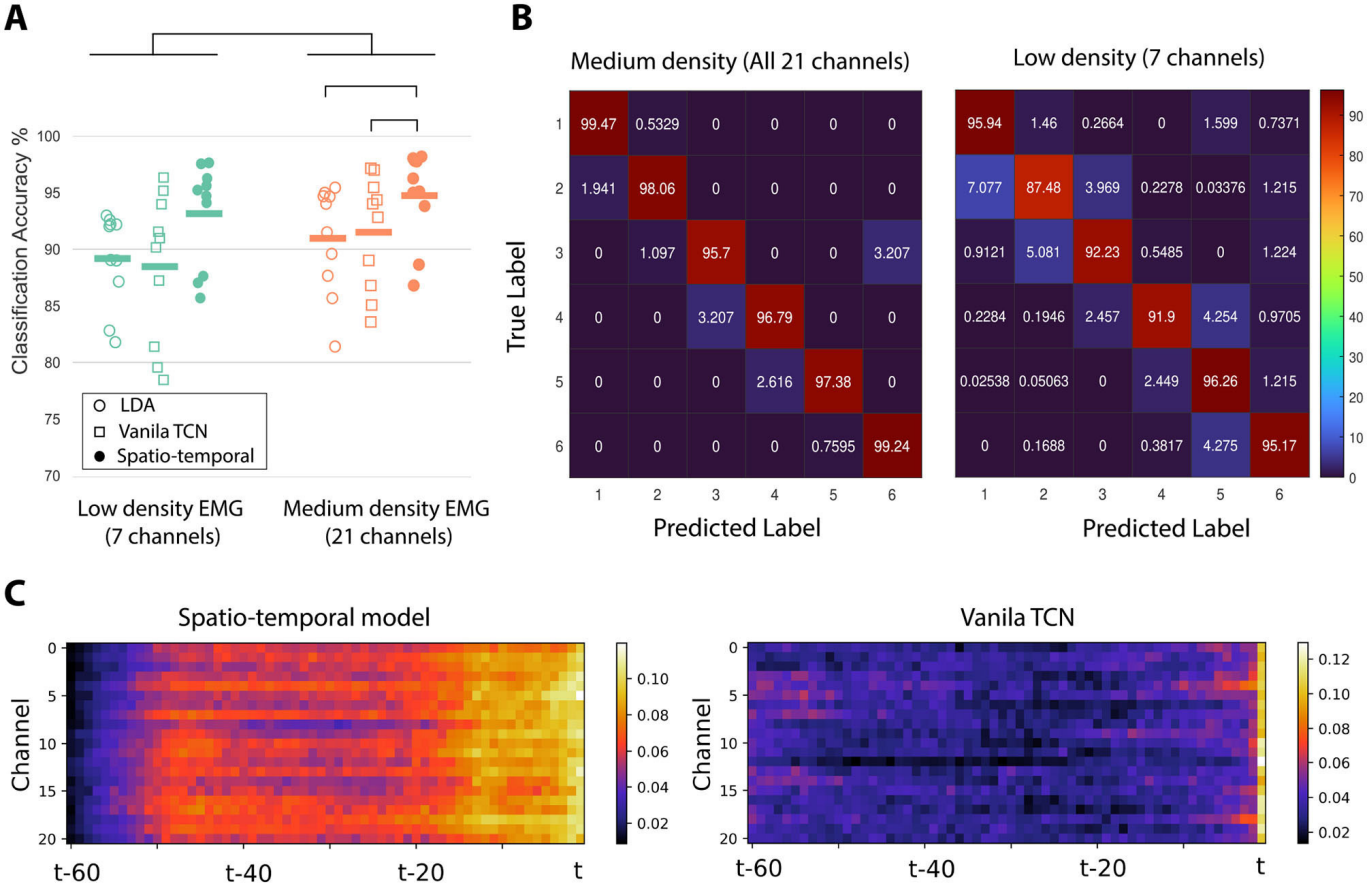
**(A)** Average classification accuracy for LDA, vanilla TCN, and spatio-temporal network when using medium density (21 channels) and low-density (7 channels) settings. **(B)** Average Confusion matrices for the same two recoding achieved using the spatio-temporal network. **(C)** Heatmap visualization of averaged saliency maps across all subjects when deploying the the spatio-temporal network and vanilla TCN.

Figure 4A demonstrates the mean accuracy achieved for each of the 10 participants for the LDA, vanilla TCN, and simultaneous spatio-temporal model for two recording configurations, (1) medium density, when the 21 EMG channel data was included and (2) low density, when only the middle EMG channel of each EMG sensing unit was included, that is, 7 channels. The results show that increasing the spatial density of the surface EMG sensors increases the classification accuracy. This finding was confirmed for statistical significance with a Wilcoxon signed rank test (*Z* = 27, *p* = 2 × 10^−6^). Furthermore, we observed a significant effect of the choice of decoder when classifying medium-density EMG data (Friedman test, χ^2^ = 12.2, *p* = 0.002, *df* = 2). *Post-hoc* pairwise analysis using a Nemenyi test revealed that the spatio-temporal model outperforms both the vanilla TCN (*p* = 0.004) and the LDA model (*p* = 0.01). The difference between the classification accuracy with the LDA and vanilla TCN models was not statistically significant (*p* = 0.97).

Figure 4B illustrates the averaged confusion matrices for all subjects, comparing the proposed simultaneous spatio-temporal network in the medium- and low-density configurations across six classes, The error distribution across all classes for the proposed model is lower than that of the Vanila TCN.

As one of the contributions in this study was to evaluate the effect of spatio-temporal feature learning, we visualized the contribution of each spatial-temporal pixel as input for both the proposed spatio-temporal model and the vanilla TCN. The heatmap representation of the saliency method used for the proposed spatio-temporal model and vanilla TCN, in the medium-density configuration, is shown in Figure 4C on the left and right, respectively. The proposed model can successfully increase the contribution of all channels simultaneously at most times, effectively addressing the spatio-temporal aspects of the EMG signals.

## 4 Discussion

This study presents a compact medium-density EMG system that improves the accuracy and usability of the decoding for myoelectric control applications. Through the development of a novel 21-channel EMG armband and deployment of spatio-temporal convolutional networks, our system achieves better performance compared to conventional low-density EMG approaches. By leveraging simultaneous spatio-temporal feature learning, our model captures long-range temporal dependencies within the EMG signals and that between different channels. The high SNR for the medium-density EMG electrodes was comparable to that obtained in previous study (Aghchehli et al., 2024) showing the quality of the proposed system. The proposed medium-density EMG system requires less complexity compared to the high-density systems that have been used for research (Jiang et al., 2021; Varghese et al., 2024), where the armband is lightweight (100g) and user-friendly, facilitating simple data collection. These findings underscore the potential of medium-density EMG systems as an alternative to traditional low- and high-density configurations for gesture recognition.

An important contribution of this study is demonstrating that increasing the number of electrodes from a low-density to a medium-density setup can improve accuracy. Saliency map interpretation reveals that this improvement is not a pure data-driven achievement and is rooted in how spatially distributed components can be extracted from a medium number of electrodes if a spatio-temporal model is well implemented. The activation of more spatio-temporal components from the input by the spatio-temporal model, compared to the vanilla TCN, which can be visually seen in Figure 4C, proves that spatial resolution may be as important as the temporal aspect for EMG-based gesture classification.

This study is a proof of concept. We acknowledge that a larger dataset is necessary to fully establish its benefits and generalisability. The observed inter-subject variability in Figure 4A highlights the need for further investigation, which we are actively pursuing in ongoing studies. Although the current sample size (*N* = 10) limits the statistical robustness, it serves as an essential step toward validating the approach. Future work will explore larger data sets, addressing machine learning performance and variability factors to refine the applicability of the method on diverse subjects.

## 5 Concluding remarks

Our findings align with emerging trends in muscle sensing technologies, particularly magnetomyography (MMG) (Zuo et al., 2020; Ghahremani Arekhloo et al., 2023, 2024). MMG measures magnetic fields generated by muscle contractions, offering several advantages over EMG, including lower susceptibility to noise and greater signal stability. The spatial resolution advancements demonstrated with our medium-density EMG system suggest that similar enhancements in MMG technology could yield even greater improvements in decoding accuracy. Studies on MMG-based decoding (e.g., Yun et al., 2024) support this perspective, indicating the potential for MMG to complement or surpass EMG in future assistive technologies (Klotz et al., 2023).

## Data Availability

The datasets presented in this study can be found in online repositories. The names of the repository/repositories and accession number(s) can be found below: https://github.com/MoveR-Digital-Health-and-Care-Hub/Mid-density-Electrodes-Dataset/.
